# Life, the Universe, and Everything: An Interview with David Haussler

**DOI:** 10.1371/journal.pgen.1003282

**Published:** 2013-01-31

**Authors:** Jane Gitschier

**Affiliations:** Departments of Medicine and Pediatrics and Institute for Human Genetics, University of California San Francisco, San Francisco, California, United States of America

Among the pantheon of computer scientists who have framed our capacity to interpret DNA sequences stands David Haussler of the University of California, Santa Cruz (UCSC). Applying his prowess in computer learning theory to the problems of protein modeling and gene structure prediction, Haussler emerged in the mid-1990s as a trail-blazer in the field of computational biology. He came to wider prominence in 2000 during the frenetic race to produce a draft sequence of the human genome by nucleating an impassioned team of coders and engineers who assembled the sequence data and launched the UCSC Genome Browser. Fittingly, his team's contribution made manifest the vision of Robert Sinsheimer, who as Chancellor of UCSC in 1985 convened a pivotal workshop to explore sequencing the human genome.

Haussler (Image 1) now plays, by my count, at least half-a-dozen leadership roles, including co-director of the Genome 10 K project, coordinating committee member of The Cancer Genome Atlas project, and director of the Center for Biomolecular Science and Engineering at UCSC. He is easily spotted by his predilection for Hawaiian shirts, whose informality, he suggests, fosters inter-disciplinary collaboration. Indeed, Haussler's ken for machine learning and his quest for the meaning of life are so expansive that I was tempted to title this piece “Deep Thought”, a nod to the fictional computer in Douglas Adams' *The Hitchhiker's Guide to the Galaxy*, but chose a more subdued allusion instead.

**Figure:**
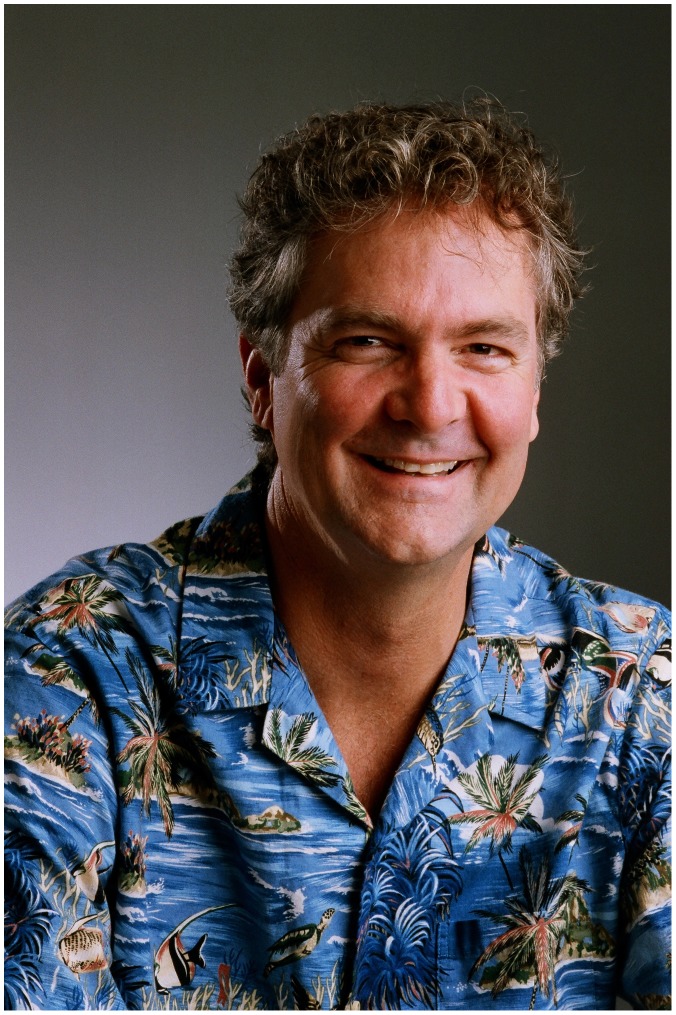
David Haussler. Photograph by Ron Jones, courtesy of the Center for Biomolecular Science and Engineering, University of California Santa Cruz.

I located Haussler on the upper reaches of the stunning UCSC campus in the engineering building, a sleek structure of glass and aluminum, tucked into a redwood grove that was still dripping and fragrant from the morning's rain. The anteroom to his modest office was decorated with handsome prints from UCSC's scientific illustration program as well as books on the genome project, and a box labeled “for the intron lounge” was piled high with journals. Haussler swept in via bicycle, swiftly signed a few documents, and downed a cold drink as we began with a discussion of his growing up in the town of North Hills in the San Fernando Valley.

**Haussler:** My dad went to Caltech and because of the economic pressures of having a young family, decided not to pursue pure science, but to pursue a professional position in structural engineering. He worked on mathematical problems as a hobbyist and had a love of pure science. Both my brother and I ended up living out his dream to be a scientist. My brother is a highly accomplished biochemist.

**Gitschier:** I saw that your first paper in the early '70s was with a Haussler and had assumed it was your father, but then, looking at his picture, I realized he must be your sibling.

**Haussler:** My only sibling is my brother. He was professor of biochemistry in University of Arizona and taught me how to do science. And he is really one of the leading scientists in the world on vitamin D, which was the subject of that first paper.

**Gitschier:** How much older is he?

**Haussler:** Twelve years.

**Gitschier:** So he was established when you were just a kid.

**Haussler:** Right. The summer after my freshman year [in college], I spent time in his lab. Every third week, I would sacrifice a chick that was raised without vitamin D. I would take out its intestines for receptors for the hormonal form of vitamin D, and we used those receptors in a radio-receptor competitive binding assay to first measure the level of the hormonal form of vitamin D in the human bloodstream, in both normal and diseased humans. By the end of the summer, we had a paper in *Science*! You know, big breakthrough.

Then I went back the next summer, and nothing worked. I remember my brother saying to me, “Now, this is how science *really* is.” But I was undaunted.

**Gitschier:** Let's talk about your transition to science, because I know your first college experience was in art.

**Haussler:** I did visual art mostly. Acrylic painting and metal sculpture were probably my favorites, although I did stone lithography and all kinds of fabulous things in the San Francisco Academy of Art. Then, I switched schools and into psychology.

**Gitschier:** And that was where?

**Haussler:** That was actually at a crazy little experimental college. You have to understand that this was the early '70s…

**Gitschier:** I do understand! [Haussler and I were born the same year.]

**Haussler:** My mother was hoping I'd go to UCLA, but I was a rebel and said “No, I want to go to a crazy place,” Immaculate Heart College [IHC] in Hollywood.

**Gitschier:** Immaculate Heart doesn't sound so “crazy” on the surface.

**Haussler:** It doesn't, not at all, but the thought leader there was Sister Corita Kent, and you remember from the '60s, those love posters? A lot of the art movement and the philosophy that was expressed in art and posters in that era actually came out of Sister Corita Kent and a number of other rebels. The sisters at IHC were essentially kicked out of the Catholic Church for being radicals, and they had an extremely experimental college. So I, being the contrarian I was, applied there. It was strong in art and music and psychology. We studied Fritz Perls and Carl Rogers and all of these self-realization psychology thinkers at the time. And I was extremely into that. We had intensive encounter groups and dug very deeply into personal interactions.

**Gitschier:** But you didn't stick with Immaculate Heart.

**Haussler:** I got interested in science by working with my brother. I then transferred to Connecticut College back east. Again, I liked very intimate, individual learning. This was part of my whole psychology background. I view essential human progress being made, including learning, within a very intensive, one-on-one or small group interaction.

**Gitschier:** When you went there, you knew you wanted to do math?

**Haussler:** Yes. During those two summers with my brother, the one thing that mattered most was not the wet lab experiments that I had done, but when it came to analyzing the data. Someone in the lab was showing concentration in relation to a radioactive response curve and trying to fit that data with a linear function. And I said, “Well you can't use linear regression on this until you transform the variables.” And they looked at me and said, “Can you do that?”

And then I realized, hey wait a minute, I can contribute on the math side and it's a lot more fun than grinding up chicken guts! I like the quote that “mathematics is the queen of sciences” [attributed to Gauss]. Mathematics is the beautiful unity in the universe, and that's what totally captivated me.

**Gitschier:** Then, you find yourself at the University of Colorado doing PhD work in computer science. That seems like a logical transition to me.

**Haussler:** Logical is the correct word. After studying pure mathematics as an undergrad, I decided that the foundation for everything was logic. And I read extensively before I went to graduate school, but even after getting my undergraduate degree in mathematics and a minor in physics, I still hadn't decided to pursue a life of science.

**Gitschier:** What were you thinking of—art, philosophy, psychology?

**Haussler:** I wanted to get at the heart of the meaning of life.

**Gitschier:** Wow. [I had to swallow the answer, “42”.]

**Haussler:** Still this rebel spirit, I guess. I wasn't convinced that I would find that at traditional institutions. I spent about nine months wandering around Europe and then settled in San Luis Obispo on the family farm, kind of between generations. My grandfather was aging and my father was active as an engineer, so there was no one to take care of it.

While I was there, I wanted to keep touch with my intellectual side, so my friends and I—it was almost like a commune—believed in working hard on the ranch during the day and then reading and discussing philosophy, history, literature, and psychology at night.

**Gitschier:** Who were these people that you recruited to the farm?

**Haussler:** Well, important people that I met in my life and in my travels. We read books and had wonderful discussions. I remember my favorite title was *The Origin of Consciousness in the Breakdown of the Bicameral Mind*. We were trying to build a non-traditional intellectual environment.

But size is a factor there. What was missing at that time was the Internet. There was no way to get in touch with other people who had very specific interests except through the library and through post. So it became a 19th century gentleman-scholar kind of activity, which has very limited impact.

**Gitschier:** What happened to the farm after you left?

**Haussler:** My father did retire there. He and my mother had a spectacular retirement, raising organic fruit and selling it at the farmers market. So I played an important role in the family; I was the bridge to that retirement and it allowed me close friendship and think time.

**Gitschier:** And what firm had your father worked for?

**Haussler:** Oh, in my family, we never worked for anybody else! Robert Haussler Structural Engineering!

**Gitschier:** I see. It was a tradition!

**Haussler:** My great grandfather, my grandfather, my father always ran their own businesses. Never had a boss. It was a crazy, fierce, independent kind of tradition.

**Gitschier:** So this was instilled in you very early. I'm now seeing the fuller context!

**Haussler:** Right. I wasn't going to play along with any institutional programs! Those were the days when you could be anti every institution and get away with it.

Well, I look back at my writings from that time and there was some very creative stuff but isolated from the bulk of the intellectual mainstream, it's very hard to make progress. So I was thrilled to get re-engaged, just by taking advanced math classes at Cal Poly [San Luis Obispo].

Applied mathematics was my major, but I took computer science classes as well. I seized on the question of what is computable. What can be formalized by mathematics? And the answer, according to Alan Turing, was that this is the same as what can be computed on a very simple kind of machine. I was tremendously taken by that and by the fact that Turing and Kurt Gödel had established that there were things that were fundamentally uncomputable; true but unprovable. It appealed to my mystical side. I was always interested in the unity of the universe and the mystery of it.

**Gitschier:** Are you still?

**Haussler:** I still am in many ways. The mystery of “why life” and “is there a mathematical inevitability that there will be life” are questions that I spend quite a bit of time thinking about. I don't write much about them because I'm engaged in areas that are more immediately applied and have urgent impact, but I think a lot about them.

And there's a theme in my thinking and in my life that has been constant since those days as a young adult searching for answers. I turned away from thinking about that as a humanistic quest—to understand my psychology and our interactions—into an absolute quest for knowledge about the universe. In a sense that is the one thread that unites my entire adult life because I've been in so many different scientific areas.

But life itself has always been something that fascinated me, life in the broadest sense, that spans everything from the actual biological life that we observe on this planet, to the abstract notion of life. Like in Conway's Game of Life where you have a disarmingly simple mathematical system that nevertheless is sufficiently complex that it is naturally an incubator of self-reproducing patterns; you start with a random pattern, and you will have emergent forms that will be self-replicating entities that interact, as in living systems.

I went through a period where I was more fascinated with what we can formalize than with the messiness of real biological life. I'm talking primarily about the act of doing science via computer. How powerful were the systems of logic and mathematical calculation in terms of revealing the nature and structure of the universe? And if we turned them loose, would they actually produce things that human minds are not able to? So I became obsessed with machine learning.

**Gitschier:** Why don't you define “machine learning”?

**Haussler:** Machine learning is the design of adaptive software systems for the purposes of modeling the world or achieving some action in response to input, as in speech recognition, in a way that embraces the complexity of the phenomena being modeled and improves with use.

**Gitschier:** And then, from there, that you'll be able to make predictions.

**Haussler:** Right. The prototypical machine learning exercise is to take a training set of examples, such as spoken utterances coupled to the actual words being spoken, and to expose a computer algorithm to that training corpus, have the computer algorithm adjust some parameters or actually invent or discover rules within those data, and then encapsulate the structure that it learns from these examples into some computer readable form. Then you test it by giving other examples where you are not supplying the answer, but asking it to predict what the answer is. Machine learning algorithms model both data as well as decisions or actions that are made in light of data.

So, I went to Colorado to study the quantitative framework for logic and mathematics that explains emergent and adaptive phenomena, like life or the complexity of systems.

**Gitschier:** And why did you choose Colorado?

**Haussler:** I chose Colorado for one reason: to work with Andrzej Ehrenfeucht. He is very broad, a polymath. And his work appealed to me very fundamentally.

If I learned from my brother how to do science, from Andrzej I learned how to think deeply and rigorously and to unleash mathematical creativity. To work with a real math genius is an intense interaction! We would spend whole Saturdays at his house, in his backyard. He would roll out the board and we would scribble on it. He also ran weekly seminars on campus, and other graduate students would attend.

**Gitschier:** I take it, Gene Myers, and also Manfred Warmuth.

**Haussler:** Yes, and don't forget Gary Stormo! Gary was one of the founders of the field of bioinformatics. He was a graduate student in molecular biology, while the rest of us were in computer science. The four of us all participated in Andrzej's weekly seminar where we would discuss any topic in science or mathematics that was exciting, and the topics were all over the map. Anybody who was engaged and wanted to could participate.

**Gitschier:** In fact, you have an early paper with Gary in 1986 about DNA sequence landscapes. This is your first foray into annotating sequences or thinking about sequences. And we're not talking hidden Markov here yet.

**Haussler:** No, this is before the hidden Markov period.

At that time, we were exposed to some very exciting data. The first DNA sequences were coming in from bacteriophage ΦX, T7, snippets of *E. coli*, and the whole recombinant DNA revolution was occurring. The language of DNA was starting to be decoded for the first time, and it seemed like an extraordinary opportunity from a mathematical point of view. Gary's thesis was on recognizing ribosome-binding sites in *E. coli* by computer analysis of DNA sequences. Meanwhile, Gene Myers had worked out the algorithms for RNA folding into its secondary structure. All of that was developed as part of Andrzej's seminar. Week after week I watched that happen and participated and kibitzed. There was no term “bioinformatics” or “genomics”. These fields weren't even named yet, but we were seeing an inkling of what could be accomplished.

I probably would have stayed with the DNA analysis had there been more of a capability to produce DNA data at the time, but months went by and there was nothing new! We looked at every scrap of DNA that existed. The field was essentially data-bound. It is interesting to think back upon that today in light of the sequencing deluge that we are now seeing!

So the bulk of my time for the next decade was really spent on the machine learning question in general.

**Gitschier:** Including after your move to Santa Cruz.

**Haussler:** Yeah, Manfred recruited me here, and he and I worked together very intensively. We, with other colleagues, Ron Rivest at MIT, Lenny Pitt who is now at University of Illinois, and several other scientists created a new scientific research group and area, the field of computational learning theory.

Here we go: COLT '88—[Haussler pulls a report from his bookshelf]—computational learning theory. This was the conference on computational learning theory and they still have them today.

The methodologies that we, the community of machine learning, developed in those decades in the '80s and '90s have come to fruition in the last decade. For example, we started with primitive speech recognition where you could speak one of ten words in a fixed vocabulary, and the speech recognition algorithm would tell you which of the ten words you spoke. Now there is Siri, and Siri doesn't come easy! Siri is based on hidden Markov models, and that framework had to be worked out and all the engineering had to be done on top of that. All of these ideas really were fermenting in the '80s and '90s. Finally, we could pull together a conceptual framework for machine learning that was much richer than had existed before.

**Gitschier:** OK. So regarding hidden Markov models, let's talk about their application to DNA. I take it that you and your post-doc Anders Krogh were really the first to start applying this model to biological problems. You have two papers in 1994—one on protein modeling and the other, which is one of my favorites, on gene prediction in *E. coli*…

**Haussler:** Oh, thank you.

**Gitschier:** …'Cause I actually understood it!

**Haussler:** Yeah, that is the first paper that applies hidden Markov models to find genes in DNA. Before this paper, there were a lot of ad hoc tools. People had independently come upon pieces of this theory, and they weren't recognizing the whole.

So the important contribution to the field here was to take what was considered a disparate toolbox of different methods and approaches and to unify it under one mathematical framework that was conceptually clean and revealed the power and the central concepts behind these methodologies. That mathematical framework—the hidden Markov model—had already been developed and is much broader than DNA or proteins; it is used very broadly in science in engineering. Again, it takes a cross-disciplinary perspective to synthesize those things.

It just struck me at one point that we really should start to think about doing this on DNA and protein sequences. And that gelled. It really snowballed very quickly. We really started to see that many things fit together. It was one of those “aha moments”.

**Gitschier:** Clearly by 1994, you are very well aware that there is a ton of sequence coming down the road.

**Haussler:** Yes, exactly. Things started cooking. The [sequencing] technology was progressing at about a Moore's law rate at that time. And it bore analogy to the computer world. It was clear that this would be a revolutionary opportunity to look at life by sequencing DNA, so we got very excited about the fact that there would be enough data for computers and machine learning to actually make a contribution to molecular biology.

It was thought in the early '80s and even the early '90s, some of the major computational contributions would be in protein structure predictions, and we were excited about that problem, but realized that it is fraught with intrinsic mathematical difficulties. I mean, that is a system that has so many degrees of freedom that it is very, very hard.

But there are other problems where you can see that extraordinary gains will be made by simply scaling them up. Hidden Markov models have the property that as you increase the amount of data, the computer time increases only linearly, not exponentially. Therefore, if the computer power is increasing exponentially, you get this enormous exponential leverage on the problem.

So we were extremely excited about the scaling, but I must say, back then, it wasn't clear at all that we would have the sequencing technology that we have today. It took an *enormou*s amount of effort to get the human genome project done. To scale from thousands of bases in the '80 s to 3 billion bases at the turn of the millennium was just an enormous technological achievement. It cost roughly $300 million just in machine time and reagents to get the first human genome. It wasn't clear where it was going to go from there.

**Gitschier:** We'll come back to that, but first, how did you come to be involved in the Human Genome Project?

**Haussler:** Eric Lander called me up in 1999 and said, “We're familiar with your work on hidden Markov models in gene finding, and we expect to have sequence for analysis sooner than we had previously planned. We would like you to participate in the analysis of it.” And of course I said, “Yes.” It was the opportunity of a lifetime to join the human genome project. I set about gathering a team, which included Jim Kent and David Kulp, who had produced a successor to his 1994 work, the GENIE program, which was used very effectively by Celera to annotate the genes in the fly genome in 1998.

**Gitschier:** OK, right. But none of this was about assembly.

**Haussler:** No. We had not done *an*y work on assembly. And we were not *asked* to do any work on assembly.

**Gitschier:** OK, so Jim Kent was a graduate student here at UCSC, and he had just produced a graphical presentation of the splice sites in *C. elegans,* right?

**Haussler:** That's right, “INTRONERATOR”.

**Gitschier:** Love that name. So Jim had already been coming up with these computer-generated representations of genes. And we're anticipating the genome browser, here.

**Haussler:** Jim's background is the computer game industry and other areas of large-scale software design, as well as biology and mathematics. He had this ability, which is unprecedented in many ways, to conceive of and quickly build software that is incredibly complicated and rich. So *that* capability was huge. It was clear that he had extraordinary talent from the beginning.

So, we were asked to work on analysis of the biological content of the DNA sequence. The problem was that the DNA sequence was in tiny pieces and the assembly efforts—there were two; they had hedged their bets—were not succeeding. So we had nothing to analyze. You can't find genes from very small pieces of DNA, even a few thousand bases is not enough. We need 50,000 bases or more at a shot.

**Gitschier:** So then you had to start addressing yourself to the assembly problem.

**Haussler:** We had to! I had a post-doc who was working on it, Nick Littlestone; his design was elegant but didn't scale enough. This was totally a skunk-works. We didn't have any funding, that wasn't our mandate, but it became clear that the whole thing would fall apart unless somebody could assemble the genome.

Meanwhile, Jim was working on issues *assuming* the assembly would work, getting more and more nervous every day that he wouldn't have any input to what he was doing unless someone did the assembly!

And at one point Jim said, “I think I'm going to do it.” And I said, “Godspeed!” And he just kicked out the code. I mean he literally worked night and day. I remember visiting him and he had to ice his wrists because he was coding so furiously!

He wrote thousands of lines of code to assemble the DNA for the first draft of the genome. And it's such a complicated problem. There were 13 different types of input that you had to solve this big constraint satisfaction problem for. Basically you had the cloned fragments, you had the genetic maps, you had the physical maps, you had RNA sequences to order and orient the fragments, all kinds of information about how the DNA should go together and much of it contradictory! You had to adjudicate those contradictions and deal with all of these different pieces of information to achieve a single assembly that made sense.

Francis Collins had prearranged with Craig Venter that we would declare a tie with Celera and a meeting was to be held at the White House to announce the first draft of the genome on June 26th, 2000. Without Jim's efforts, it never would have happened! And it is such a complicated and tricky problem that a committee could argue about it for years, or a set of programmers could design a modular approach and each solve part of the problem, which would take years to implement! Only one person, a genius with everything in his head and incredible skills would pull this off! And he did it in about four weeks! I mean, it was unprecedented.

**Gitschier:** He just started with this problem…

**Haussler:** …And did it.

Jim delivered the assembly on June 22nd, and then we spent a weekend furiously analyzing it. You know, Francis was on the email or the phone to everybody and we were going back and forth. The entire international team had to analyze it. And we found out from Gene Myers, who was our counterpart from Celera, that his team finished on June 24th, two days before the conference, and was going through exactly the same experience analyzing the Celera draft of the human genome.

Later, we had the advantage, of course, because Celera couldn't really publicly unveil its genome; you had to pay a subscription for it. But we could, so we had the honor of posting it from UCSC on the Internet on July 7th. Now that was *really* the greatest moment.

**Gitschier:** Now, when you posted it, it was in the framework of a genome browser?

**Haussler:** No, at that point it was raw DNA.

**Gitschier:** Oh, God. So when did the browser become official?

**Haussler:** So that was shortly after that. Jim took the INTRONERATOR code and turned around and made the UCSC Human Genome Browser, and to make a long story short, that's been a huge success.

**Gitschier:** OK, and now let's return to this exponential growth in sequencing capability since then.

**Haussler:** Really, the “hyper” Moore's Law [for DNA sequencing] started in about 2005, 2006, where suddenly we're on a curve where DNA sequencing is improving ten times every two years. So you have to think about these numbers! Computers continue to go along at most at two times improvement every two years, so for the last eight years, that's 2×2×2×2, well that's great! Computers are 16-fold more powerful! DNA sequencing: 10×10×10×10! That is 10,000 times more powerful! It's *huge*!

It's incredibly disruptive technology. It will affect everyone's lives! It's very seldom that you have this kind of curve. I can't think of another technology like this. For me, it's an amazing experience, to remember the days when we would wait *years* for another few thousand bases to appear—to this! The cumulative effects of sequencing technology improvements are just unbelievable! It's unbelievable! When you have such a steep exponential growth law, you get a shocking change on time scales that are a blink of an eye in terms of science and society.

